# The Efficacy and Cost-Effectiveness of Cell Saver Use in Instrumented Posterior Correction and Fusion Surgery for Scoliosis in School-Aged Children and Adolescents

**DOI:** 10.1371/journal.pone.0092997

**Published:** 2014-04-01

**Authors:** Yu-Liang Miao, Hua-Song Ma, Wen-Zhi Guo, Ji-Gong Wu, Yan Liu, Wen-Zhu Shi, Xiao-Ping Wang, Wei-Dong Mi, Wei-Wu Fang

**Affiliations:** 1 Anesthesia and Operation Center, Chinese PLA General Hospital, Beijing, P.R. China; 2 Department of Anesthesiology, Chinese PLA No. 306 Hospital, Beijing, P.R. China; 3 Department of Orthopaedics and Spine Surgery, Chinese PLA No. 306 Hospital, Beijing, P.R. China; 4 Department of Anesthesiology, Beijing Military General Hospital of the PLA, Beijing, P.R. China; German Red Cross Blood Service Frankfurt, Germany

## Abstract

Posterior spinal instrumentation and fusion surgery in school-aged children and adolescents is associated with the potential for massive intraoperative blood loss, which requires significant allogeneic blood transfusion. Until now, the intraoperative use of the cell saver has been extensively adopted; however, its efficacy and cost-effectiveness have not been well established. Therefore, the aim of this study is to determine the efficacy and cost-effectiveness of intraoperative cell saver use. This study was a single-center, retrospective study of 247 school-aged and adolescent patients who underwent posterior spinal instrumentation and fusion surgery between August 2007 and June 2013. A cell saver was used intraoperatively in 67 patients and was not used in 180 patients. Matched case-control pairs were selected using a propensity score to balance potential confounders in baseline characteristics. Allogeneic red blood cell (RBC) and plasma transfusions as well as blood transfusion costs were analyzed. The propensity score matching produced 60 matched pairs. Compared to the control group, the cell saver group had significantly fewer intraoperative allogeneic RBC transfusions (*P* = 0.012). However, when the combined postoperative and total perioperative periods were evaluated for the use of allogeneic RBC transfusion, no significant differences were observed between the two groups (*P* = 0.813 and *P* = 0.101, respectively). With regard to the total cost of perioperative transfusion of all blood products (RBC and plasma), costs for the control group were slightly lower than those of the cell saver group, but this variance did not reach statistical significance (*P* = 0.095). The use of the cell saver in posterior spinal instrumentation and fusion surgery in school-aged children and adolescents was able to decrease the amount of intraoperative allogeneic RBC transfusion but failed to decrease total perioperative allogeneic RBC transfusion. Moreover, the use of the cell saver was not cost-effective.

## Introduction

Primary posterior spinal instrumentation and fusion surgery for scoliosis is a major spinal operation that is associated with massive intraoperative blood loss [1], [2]. Over the past decades, despite significant improvements in the safety of allogeneic RBC transfusion, there remain underlying risks such as allergic reactions and the risks of bacterial, malarial, HIV and hepatitis infections [3]. Furthermore, allogeneic RBC transfusion may be related to an increased rate of operative site infections [4]. Should blood transfusion complications ensue in children and adolescents, the consequences may be serious and long-term.

In recent years, to decrease the need for allogeneic blood transfusion, alternative approaches such as the use of the cell saver, which can salvage and return the patient's RBCs, have been widely used intraoperatively [3]. Use of the cell saver has extended to spinal deformity surgery, including surgery for scoliosis. However, there are conflicting reports about the efficacy and cost-effectiveness of intraoperative use of the cell saver. Some studies have indicated that use of the cell saver decreased RBC transfusions in scoliosis surgery [5]–[7], while others have demonstrated that cell saver use presented little benefit or might have been associated with increased blood loss [8]–[11]. In all, few studies have evaluated the intraoperative use of the cell saver in a younger age group such as ours.

Therefore, we designed this retrospective study to analyze a large series of school-aged and adolescent patients undergoing elective posterior spinal pedicle screw instrumentation and fusion surgery to determine the efficacy and cost-effectiveness of the intraoperative use of the cell saver.

## Materials and Methods

The study was a retrospective review of charts and anesthesia records of patients at the People's Liberation Army (PLA) hospital No. 306 and was approved by the institutional review board and the ethics committee of the hospital No. 306. Since this was a retrospective sutdy and the information of the patients was anonymized, no written informed consent was obatined from the participants. Chart review was performed by 5 authors (Y.-L. Miao, W.-W. Fang, W.-Z. Shi, Y. Liu and W.-Z. Guo). A total of 247 consecutive patients with scoliosis who underwent primary posterior instrumentation and fusion surgery between August 2007 and June 2013 were enrolled. At our hospital, the use of the cell saver is not considered in patients younger than 7 years of age or in patients whose weight is less than 20 kg. Therefore, the study inclusion criteria were patients between 7 and 18 years of age who weighed more than 20 kg and underwent primary posterior pedicle screw instrumentation and spinal fusion surgery for scoliosis through a midline approach. Exclusion criteria included patient age younger than 7 or older than 18 years, body weight less than 20 kg, history of a clotting disorder and a platelet count lower than 100,000/mm^3^.

Patients' charts and anesthesia records were thoroughly queried from the electronic medical record database to collect the following clinical and diagnostic data: gender; age; weight; pre- and postoperative Cobb angle of the major curvature; number of spinal levels fused; duration of the operation (minutes); intraoperative use of the cell saver; intraoperative estimated blood loss (EBL); volume of the postoperative drainage; volume of the cell saver collection that was reinfused; the preoperative and discharge hemoglobin (Hb) and hematocrit (Hct) levels; and, most importantly, amount/volume of intraoperative and postoperative allogeneic RBCs and fresh-frozen plasma (FFP) transfused.

The Department of Orthopedics of the PLA hospital No. 306 is one of the spine and joint surgery centers of the PLA, with a dedicated team of spinal surgeons who perform approximately 1000 spinal operations per year. Moreover, it was one of the first institutions in China (1999) to employ posterior spinal pedicle screw instrumentation and fusion techniques for the correction of scoliosis. On average, more than 200 patients afflicted by various types of scoliosis are admitted to this center for surgical intervention every year.

The cell saver has been used from August 2006 to the present; however, the electronic medical record system was introduced at our institution in January 2007. Detailed clinical information on scoliosis patients before this time point was not optimally recorded and was difficult to review. With allowance for a “grace period” of 6 months after the introduction of the electronic medical record system, the study cohort of patients included those who were accepted as surgical candidates between August 2007 and June 2013. The patients were classified according to use of the cell saver into a control group and a cell saver group.

### Surgical approaches

The majority of the operations were performed by two authors (H.-S. Ma & J.-G. Wu) who have been dedicated to this field for more than fifteen and ten years. The patients underwent cervical and/or thoracic and/or lumbar laminectomy and arthrodesis according to the standard posterior approach. If indicated, osteotomy and hemivertebral resection were performed. Instrumentation was achieved using segmental pedicle screws. Posterolateral vertebral plate decortication was accomplished using a high-speed burr and rongeurs, followed by autogenous iliac crest and/or rib and/or allogeneic bone graft that was placed as dictated by the specific circumstances of the individual procedure. One drain was routinely placed under the muscles of the back before closure of the incision to allow continuous suction, and it was withdrawn no later than postoperative day three, when the drainage volume was less than 100 ml per day.

The decision to use or not use the cell saver was made by the surgeons, who tended to use the cell saver for cases in which major blood loss was anticipated. The “cell saver” cohort all had intraoperative cell salvage performed by an experienced technician in accordance with the manufacturer's guidelines. The washed RBCs were reinfused during the intraoperative period. For cases when the cell saver blood was insufficient, complementary allogeneic blood use was indicated. The intraoperative EBL was determined based on the combined volume of blood gathered in the cell saver canister, suction canisters and swab wash. The postoperative EBL was determined by measuring the amount of blood in the drainage bag. The total perioperative amount of EBL was calculated. The intra- and total perioperative blood replacement was described as % of (calculated) blood volume which was set at 70 mL/kg (weight).

According to the formal transfusion protocol of the PLA hospital No. 306, intraoperative indications for transfusion were signs of anemia such as hypotension that was inadequately responsive to fluid challenge; blood loss greater than 20% of the total blood volume; and a hemoglobin level less than 8.0 g/dl or an absolute hemoglobin level of less than 7.0 g/dl without signs of anemia. The same transfusion guidelines were followed during the postoperative period. Coagulopathy is believed to develop as a consequence of massive blood loss and massive red cell transfusion. Therefore, fluid management approaches emphasize the use of an adequate volume of FFP simultaneously with platelet transfusions. However, there was no formal protocol concerning the transfusion of FFP at our hospital. Thus, the amount of FFP transfused was determined by the combined discretion of the surgeon and the anesthesiologist.

The main anesthetic approach is summarized as follows. General anesthesia was induced by intravenous midazolam 0.1 mg/kg, fentanyl 2 μg/kg, propofol 2–3 mg/kg and vecuronium 0.1 mg/kg. The patient was intubated with an armored endotracheal tube. Anesthesia was maintained by continuous infusion of propofol and remifentanyl and intermittent injections of fentanyl and vecuronium. Continuous ECG, pulse oximetry, end-tidal CO_2_, invasive arterial blood pressure (ABP), central venous pressure (CVP), urine output and blood-gas analysis were routinely monitored. All of the patients underwent wake-up testing, and some of the patients accepted the use of somatosensory evoked potential monitoring to avoid possible spinal neural damage when the scoliosis was corrected.

No patient donated autologous blood preoperatively, no hemodilution technique was employed, and no procoagulant medicine was administered during the operative procedures. Deliberate hypotension was achieved by modulating the anesthesia depth to maintain a mean arterial pressure (MAP) between 40 and 50 mmHg.

At our hospital, the cost for transfusion of each unit of allogeneic packed RBCs is $70.49, which includes ABO and Rh blood typing, antibody screening, cross matching, packed RBCs, white blood cell filtration and administrative expenses (Table 1). The cost for the use of the cell saver is a flat rate charge of $311, which includes tubing, liner and anticoagulant solution costs. The cost for each package of FFP (200 ml), including administrative expenses, is $13. The total transfusion cost for every patient was calculated.

**Table 1:** Allogeneic blood cost.

Expenditure	Cost ($)
ABO and Rh typing	3.28
Antibody screen	14.75
Cross match	9.84
Packed RBCs	34.43
White blood cell filtration	6.89
Administration	1.31
Total	70.49

### Statistics

The two groups were compared for preoperative baseline and perioperative factors. Because patients were not randomized by gender, age, weight, preoperative Cobb angle, preoperative Hb and Hct, EBL, duration of the operation and number of spinal levels fused, we attempted to reduce the bias due to confounding by using propensity score matching [12], [13].

A logistic regression model was created with a stepwise option to derive a propensity score that included parameters available about the patients and the surgical procedures. Each patient was assigned a propensity score that reflected the intraoperative EBL. The variables tested in the propensity score were gender, age, body weight, preoperative Cobb's angle of major curvature, EBL, preoperative Hb and Hct, duration of the surgery and number of spinal levels fused. Based on the propensity score of each patient, a 1∶1 matched and 0.2 caliper analysis was performed without replacement. The aim of this procedure was to attempt to mimic randomization by creating 2 groups of patients who were comparable with respect to all covariates mentioned above.

Each patient in the cell saver group was matched with the patient in the control group with the closest propensity score using an 8-1 digit match algorithm [12]. Patients who did not have close pairings were excluded from the final matched population.

Finally, the groups were compared according to perioperative parameters with the primary outcome parameters determined as intraoperative and postoperative allogeneic transfusion amount, total perioperative transfusion amount and the cost of all blood transfusion-related expenses. Categorical variables were summarized as frequencies and continuous variables as the mean ± standard error of the mean (SEM). Characteristics of the 2 groups were compared by using Student's-t test for continuous variables and the chi-square test for categorical variables. All statistical analyses were performed using Statistical Package for the Social Sciences (IBM SPSS Statistics 19.0, Chicago, IL,USA), and the statistical significance was defined as *P*<0.05.

## Results

After applying the exclusion criteria, a total of 247 patients initially qualified for our study. The cohort consisted of 67 patients in whom the cell saver was used and 180 patients in whom no cell saver was used. Demographic characteristics and perioperative factors regarding the patients before and after matching are presented in Table 2.

**Table 2:** Patients' demographic characteristics and perioperative factors, before and after propensity score matching.

	Overall Cohort			Propensity-Matched Cohort		
	Control	Cell saver		Control	Cell saver	
	(n = 180)	(n = 67)	*P*	(n = 60)	(n = 60)	*P*
Gender (Male/Female)	65/115	27/40	0.545	23/37	22/38	0.850
Age (yr)	13.89±0.21	14.57±0.28	0.072	14.93±0.31	14.65±0.28	0.496
Body weight (kg)	38.09±0.77	43.39±1.35	<0.001	42.04±1.30	42.13±1.28	0.960
Pre-op hemoglobin (g/dl)	126.85±1.03	130.84±1.85	0.049	128.62±1.70	130.01±1.99	0.597
Pre-op hematocrit	40.11±0.43	40.57±1.03	0.624	39.83±0.59	40.44±1.14	0.632
Pre-op Cobb angle of major curvature(°)	87.66±2.13	91.40±3.60	0.365	94.70±3.31	90.40±3.88	0.401
Intraoperative EBL (ml)	1541.94±82.72	2352.99±169.02	<0.001	2135.83±168.33	2185.83±169.82	0.835
Duration of the operation (min)	298.96±6.14	337.87±8.91	0.001	334.67±11.01	328.62±8.82	0.669
Number of levels fused	10.18±0.31	10.03±0.494	0.803	9.88±0.501	9.92±0.537	0.964

The propensity score matching produced 60 matched pairs. No match was found for 7 patients in the cell saver group because there were insufficient patients in the control group with the proper matching score. Between the two groups, matching was satisfactory in achieving balance with respect to the variables we collected, in particular for the most important factor: intraoperative EBL (Table 2). No serious complications concerning the transfusion of autogenic or allogeneic blood were reported, and no blood coagulation disorders were encountered.

There were 59 patients in the control group and 58 in the cell saver group who received allogeneic RBC transfusion. A comparison of the transfusion findings and other perioperative results are presented below (Table 3).

**Table 3:** Transfusion tally and other perioperative results for propensity-matched cohort.

	Control	Cell saver	*P*
	(n = 60)	(n = 60)	
Allogeneic RBC transfusion (units)			
Intraoperative (units)	8.28±0.54	6.40±0.51	0.012
Postoperative (units)	3.63±0.62	3.45±0.46	0.813
Total perioperative (units)	11.92±0.93	9.85±0.78	0.101
FFP transfusion (ml)			
Intraoperative (ml)	620.00±48.18	540.00±59.24	0.279
Postoperative (ml)	293.33±97.13	235.00±42.44	0.583
Total perioperative (ml)	913.33±117.16	775.00±81.88	0.335
Other perioperative parameters			
Crystalloids (ml)	1650.91±92.91	1547.46±70.29	0.372
Colloids (ml)	1422.81±93.32	1474.58±79.65	0.673
Reinfused amount of RBC (ml)		561.63±51.74	
Duration of ICU stay (h)	6.29±0.95	5.76±0.73	0.659
Postoperative hospital stay (days)	17.53±1.29	17.53±0.77	0.996
Postoperative drainage (ml)	852.92±109.34	769.21±87.95	0.567
Total perioperative amount of EBL (ml)	2889.25±215.37	2724.28±211.49	0.586
Intraoperative blood replacement (%)	77.25±6.82	81.38±7.98	0.695
Total blood replacement (%)	104.69±9.15	101.47±9.94	0.812
Postoperative Cobb angle of major curvature (°)	41.89±3.57	38.11±2.51	0.376
Discharge hemoglobin (g/dl)	104.58±2.86	98.73±3.39	0.191
Discharge hematocrit	31.87±0.96	30.80±1.19	0.485

During the intraoperative period, the amount of allogeneic RBCs transfused in the control group was significantly higher than that in the cell saver group (8.28±0.54 vs 6.40±0.51 units, *P* = 0.012). However, the amount of FFP transfused between the groups was not significantly different (620.00±48.18 *vs* 540.00±59.24 ml, *P* = 0.279).

During the postoperative period, the amount of allogeneic RBC transfused in the control and cell saver groups was not significantly different (3.63±0.62 vs 3.45±0.46 units, *P* = 0.813). The amount of FFP transfused in each group was also not significantly different (293.33±97.13 *vs* 235.00±42.44 ml, *P* = 0.583).

When the observation period was examined in its entirety, the amount of perioperative total allogeneic RBC units transfused for each patient in the control group and the cell saver group did not significantly differ (11.92±0.93 vs 9.85±0.78 units, *P* = 0.101). Moreover, the volume of FFP transfused in the two groups was also not significantly different (913.33±117.16 *vs* 775.00±81.88 ml, *P* = 0.335).

Furthermore, there were no significant differences with regard to intraoperatively transfused crystalloids and colloids (*P* = 0.372 and *P* = 0.673, respectively), the duration of patient stay in the ICU (*P* = 0.659), postoperative hospital stay (*P* = 0.996), postoperative drainage (*P* = 0.567), total perioperative EBL (*P* = 0.586), intraoperative blood replacement (*P* = 0.695), total blood replacement(*P* = 0.812), postoperative Cobb angle of major curvature (*P* = 0.376), discharge Hb and Hct (*P* = 0.191 and *P* = 0.485, respectively).

The total cost of perioperative transfusion of all blood products (including reinfused RBCs, allogeneic RBCs and FFP) was calculated for each patient. According to the costs standard of our hospital, the total expenses for blood products in the control group were slightly lower than in the cell saver group, but the marginal variance did not reach statistical significance ($ 899.92±72.07 vs 1056.64±58.95, *P* = 0.095). However, the costs of allogeneic blood products in China are relatively low (RBC/plasma = $70.49/$13) compared to the approximate costs of US (RBC/plasma = $250/$75). The packed RBC concentrate is the equivalent of 200 ml of blood in China and 500 ml in US. Therefore, assuming the cost of cell saver use is the same in China and US ($ 311), under the premise of US standard the total transfusion cost for every patient was calculated. Under this standard, the total expenses for blood products in the control and cell saver groups was not significantly different ($1534.17±131.79 vs 1586.63±103.54, *P* = 0.775).

## Discussion

This study examined perioperative blood loss and its management in school-aged children and adolescents undergoing posterior correction of scoliosis with instrumentation and fusion. For many years, instrumented posterior correction has been considered major spine surgery and has been associated with significant blood loss that often requires blood replacement. During the past decades, despite improvements in laboratory test methods and careful screening of donated blood that have decreased the incidence of blood-borne, transfusion-related infectious diseases, assurances of complete safety from transmissible diseases could not be achieved till now [14].

The cell saver has been used clinically for decades and has been widely applied in contemporary spinal surgery. However, current reports in the literature point to conflicting points of view regarding the efficacy and cost-effectiveness of its use.

Some authors have demonstrated that the cell saver did not decrease allogeneic blood transfusion requirements in spine surgery studies performed in adults. Owens et al [8] demonstrated in adult posterolateral fusion surgery patients that the use of autologous cell saver transfusion did not reduce requirements for intraoperative or postoperative allogeneic blood transfusion. Canan et al [9] indicated that the use of the cell saver for single-level posterior lumbar decompression and fusion did not significantly reduce the need for allogeneic blood transfusion and was not cost-effective. In a systematic review, Elgafy et al [10] noted that there was little support for routine use of the cell saver in major elective spinal surgery with regard to safety and efficacy considerations. Furthermore, similar findings have been reported for adolescent spinal surgery. Weiss et al [11] demonstrated that in patients undergoing spinal fusion for scoliosis, the use of the cell saver did not decrease the rate of allogeneic transfusion.

However, apart from these negative studies suggesting that use of the cell saver is expensive and ineffective, other studies support use of the cell saver. Bowen et al [5] demonstrated that cell saver use decreased allogeneic transfusion, particularly in operations >6 hours in duration with an estimated blood loss >30% of the total blood volume in pediatric idiopathic scoliosis patients. Ersen et al [6] reported that the cell saver reduced both intra- and postoperative blood transfusion in patients undergoing posterior spinal fusion for adolescent idiopathic scoliosis. A systematic review by Carless et al [7] demonstrated that the cell saver was efficacious in reducing the need for allogeneic red cell transfusion in adult elective orthopedic surgery.

The preoperative Cobb angle of major curvature in our study was 93° and ranged from 50–65°in similar studies [5], [6], [11]. Intraoperative EBL in our study was approximately 2161 ml, while that in similar studies ranged between approximately 700 and 1100 ml [5], [6], . There are several possible reasons for these findings. First, China has the largest population in the world, and with an incidence of scoliosis of approximately 1.06% [17]. therefore China has a vast number of such patients. Second, China is a developing country, and economic conditions vary greatly between different provinces and different families. The poor economic conditions among scoliosis patients have limited disease prevention and early treatment, and therefore, the pathogenesis of this condition tends towards progression to a serious stage. Finally, our hospital is a high volume center of excellence that has a dedicated team of spinal surgeons and anesthesiologists who have participated in the instrumented posterior correction of scoliosis from 1999. Thus, many seriously ill patients have been transferred from other institutions to our center.

Of note, the results of our study are in partial agreement with the findings of previous studies that supported the use of the cell saver as efficacious. In our study, there were less intraoperative allogeneic blood transfusions in the cell saver group (*P* = 0.012), but during the postoperative period, there was no significant difference between the control and cell saver groups with regard to transfusion requirements (*P* = 0.813). Finally, if we calculate overall perioperative allogeneic blood transfusions, there was no significant difference between the two groups (*P* = 0.101). An important finding in our study is that to date, few studies have reported on the transfusion of fresh-frozen plasma, which must be transfused in adequate quantities when the patient accepted massive red cell transfusion to avoid possible coagulopathy. In our study, there were no significant differences in intra-, post- and total perioperative FFP transfusion between the control and cell saver groups.

For any clinical study, baseline characteristics are one of the most important preconditions for data analysis and result reliability. Unfortunately, the baseline conditions and characteristics of age, weight, preoperative Cobb angle of major curvature and EBL, perhaps the most important factor, were significantly imbalanced or not available in some previous studies [5], [6], [9], [11], [16], [18]. Therefore, their results and conclusions may be drawn into question.

To our knowledge, our study investigated the largest population of this type to date. Moreover, this is the first report to use the statistical method of propensity score matching to diminish the bias between the control and cell saver groups. In our study, all patients accepted a similar anesthesia method and blood transfusion protocol. No other methods of blood management were used in any patients. Therefore, only the isolated effect of cell saver use on blood transfusion was examined.

In terms of the cost-effectiveness analysis, when we calculated the total cost of perioperative transfusion of all blood products, the cost in the control group was slightly lower than that in the cell saver group, but the marginal difference was not significant (*P* = 0.095). Therefore, from a health economics stance, we concluded that cell saver use is not cost-effective.

The subjects in our study are children and adolescents, and their weights, % intraoperative blood loss and preoperative Cobb angles vary considerably. For example, one liter blood lose in a small child means exsanguination and in a large one is irrelevant. Therefore, we ranked the 120 patients from the lowest to the highest according to the three factors, respectively. Then we divided them into three groups with 40 cases in each subgroup (low, intermediate and high) to further investigate whether the cell saver could be efficacious and cost-effective in each group. The result is that cell saver was not efficacy and cost-effectiveness in any of the groups (as shown in tables S1–S3 in File S1). On the other side, cell saver decreased the need for intraoperative allogeneic RBC transfusion even though it didn't decrease the need in post- and perioperative period. This is valuable to our clinical approaches, because use of cell saver alleviated the contradiction of massive blood demand in the operative day.

There are some limitations to our study. First, the diagnoses of scoliosis encompass idiopathic scoliosis, congenital scoliosis and neuromuscular scoliosis such as cerebral palsy and muscular dystrophy, among others [19]. Second, in some patients, surgical procedures such as osteotomy and corpectomy can lead to major bleeding. Although we achieved a satisfactory balance of the baseline characteristics between the control and cell saver groups by using the propensity score matching method, the distribution of these factors was not investigated in further detail between the groups. Third, the costs of allogeneic blood products in China are relatively low compared to the US and European standards whilst the use of cell saver is comparably expensive (>300 $), furthermore, the costs associated with blood transfusion comprises of more variables [20]. However, these factors were not examined thoroughly. Finally, our study design was retrospective and was performed at a single-center.

## Conclusions

In posterior spinal instrumentation and fusion surgery for scoliosis in school-aged children and adolescents, the use of the cell saver decreased the need for intraoperative allogeneic red blood cell transfusion but failed to decrease total perioperative allogeneic red blood cell transfusion. From the standpoint of health economics, the use of the cell saver is not cost-effective. Therefore, there is a need for additional research to include adequately powered, high-quality randomized controlled studies to further investigate the use of the cell saver.

## Supporting Information

File S1 **Supporting information tables. Table S1, Main outcomes divided by weight. Table S2, Main outcomes divided by % blood loss. Table S3, Main outcomes divided by preoperative Cobb angle (°).**
(DOC)
