# Supplementary material for: The Efficacy and Cost-Effectiveness of Cell Saver Use in Instrumented Posterior Correction and Fusion Surgery for Scoliosis in School-Aged Children and Adolescents
Source: PLoS One. 2014 Apr 1;9(4):e92997. doi: 10.1371/journal.pone.0092997 (PMC3972210; doi:10.1371/journal.pone.0092997)
Supplement: File S1 — Supporting information tables. Table S1, Main outcomes divided by weight. Table S2, Main outcomes divided by % blood loss. Table S3, Main outcomes divided by preoperative Cobb angle (°). (DOC) [file pone.0092997.s001.doc]

**Supplemental materials for PONE-D-13-53635**

**The efficacy and cost-effectiveness of cell saver use in instrumented posterior correction and fusion surgery for scoliosis in school-aged children and adolescents**

Yu-Liang Miao^1,2☯^, Hua-Song Ma^3☯^, Wen-Zhi Guo^4☯^, Ji-Gong Wu^3^, Yan Liu^2^, Wen-Zhu Shi^1^, Xiao-Ping Wang^3^, Wei-Dong Mi^1*^, Wei-Wu Fang^2*^

Table S1: Divided by weight

|  | **weight** | | | | | | | | |
| --- | --- | --- | --- | --- | --- | --- | --- | --- | --- |
|  | low | | | intermediate | | | higher | | |
|  | control group | saver group | *P* | control group | saver group | *P* | control group | saver group | *P* |
|  | (n=21) | (n=19) |  | (n=20) | (n=20) |  | (n=19) | (n=21) |  |
| Age | 12.95±0.51 | 14.05±0.47 | 0.122 | 15.75±0.41 | 14.05±0.46 | 0.009 | 16.26±0.37 | 15.76±0.39 | 0.361 |
| Body weight (kg) | 30.60±1.04 | 30.74±1.33 | 0.933 | 43.75±0.48 | 42.65±0.53 | 0.130 | 52.89±1.09 | 51.95±1.05 | 0.538 |
| % intraoperative blood loss | 101.53±14.17 | 111.85±19.05 | 0.662 | 69.24±10.74 | 60.73±8.09 | 0.531 | 58.99±7.15 | 70.64±8.68 | 0.313 |
| % total blood loss | 141.33±20.35 | 137.11±23.40 | 0.892 | 87.77±10.72 | 80.59±10.81 | 0.640 | 82.16±10.33 | 85.14±10.96 | 0.845 |
| Cobb angle（°） | 95.24±7.63 | 101.79±6.43 | 0.520 | 94.90±4.28 | 87.30±6.99 | 0.359 | 93.89±4.67 | 83.05±6.31 | 0.183 |
| amount of blood recovered with the cell saver |  | 605.84±94.77 |  |  | 427.65±69.23 |  |  | 649.24±97.91 |  |
| % of lost blood recovered with the cell saver |  | 29.13±4.99 |  |  | 14.47±2.36 |  |  | 18.27±2.83 |  |
| $ total cost (China standard) | 993.04±165.31 | 1195.41±124.64 | 0.342 | 859.76±81.39 | 964.29±104.58 | 0.435 | 837.55±109.16 | 1016.36±73.15 | 0.174 |
| $ total cost (US standard) | 1713.10±318.96 | 1797.84±216.48 | 0.831 | 1448.75±131.85 | 1427.25±186.69 | 0.926 | 1426.32±180.25 | 1547.31±131.76 | 0.586 |
| Intraoperative EBL (ml) | 2138.10±308.875 | 2231.58±333.864 | 0.838 | 2095.00±307.66 | 1797.50±238.65 | 0.450 | 2176.32±266.02 | 2514.29±296.83 | 0.406 |
| Total perioperative amount of EBL (ml) | 2977.62±427.249 | 2759.84±417.390 | 0.718 | 2664.75±309.70 | 2376.50±311.01 | 0.515 | 3027.89±380.61 | 3023.33±370.58 | 0.993 |

Table S2: Divided by % blood loss

|  | **% blood loss** | | | | | | | | |
| --- | --- | --- | --- | --- | --- | --- | --- | --- | --- |
|  | low | | | intermediate | | | higher | | |
|  | control group | saver group | *P* | control group | saver group | *P* | control group | saver group | *P* |
|  | (n=19) | (n=21) |  | (n=21) | (n=19) |  | (n=20) | (n=20) |  |
| Age | 15.32±0.472 | 13.71±0.421 | 0.015 | 14.76±0.621 | 14.79±0.487 | 0.973 | 14.75±0.528 | 15.50±0.44 | 0.281 |
| Body weight (kg) | 45.63±2.100 | 43.33±1.721 | 0.399 | 42.57±2.14 | 43.84±2.39 | 0.693 | 38.08±2.29 | 39.25±2.42 | 0.726 |
| % intraoperative blood loss | 28.93±2.54 | 30.42±2.15 | 0.656 | 66.01±27.16 | 69.88±3.40 | 0.375 | 135.10±11.05 | 142.84±13.65 | 0.662 |
| % total blood loss | 46.91±4.31 | 39.69±3.69 | 0.208 | 89.53±4.42 | 89.23±6.77 | 0.971 | 175.66±17.33 | 173.79±17.13 | 0.939 |
| Cobb angle（°） | 83.37±5.22 | 83.71±6.73 | 0.968 | 95.67±4.89 | 88.05±5.35 | 0.299 | 104.45±6.31 | 99.65±7.55 | 0.629 |
| amount of blood recovered with the cell saver |  | 302.90±29.63 |  |  | 471.32±51.39 |  |  | 919.10±103.72 |  |
| % of lost blood recovered with the cell saver |  | 10.22±1.09 |  |  | 16.50±1.99 |  |  | 34.92±4.51 |  |
| $ total cost (China standard) | 617.08±74.95 | 652.33±44.75 | 0.682 | 774.21±46.15 | 1125.26±82.32 | <0.001 | 1928.98±166.28 | 1413.16±89.79 | 0.549 |
| $ total cost (US standard) | 1042.11±123.04 | 849.10±73.12 | 0.176 | 1297.62±76.24 | 1699.82±141.04 | 0.014 | 2250.00±315.58 | 2253.50±148.17 | 0.992 |
| Intraoperative EBL (ml) | 928.95±84.23 | 897.62±62.34 | 0.764 | 1947.62±119.65 | 2078.95±97.51 | 0.406 | 3480.00±268.09 | 3640.00±228.54 | 0.652 |
| Total perioperative amount of EBL (ml) | 1474.47±128.61 | 1160.00±86.62 | 0.046 | 2631.43±174.88 | 2642.11±176.99 | 0.966 | 4504.00±377.38 | 4444.85±298.02 | 0.903 |

Table S3: Divided by % preoperative Cobb angle ( ° )

|  | **Pre-op Cobb angle of major curvature（°）** | | | | | | | | |
| --- | --- | --- | --- | --- | --- | --- | --- | --- | --- |
|  | low | | | intermediate | | | higher | | |
|  | control group | saver group | *P* | control group | saver group | *P* | control group | saver group | *P* |
|  | (n=21) | (n=19) |  | (n=16) | (n=24) |  | (n=23) | (n=17) |  |
| Age | 14.71±.49 | 14.05±.554 | 0.379 | 15.00±0.68 | 14.75±0.40 | 0.738 | 15.09±0.51 | 15.18±0.46 | 0.900 |
| Body weight (kg) | 41.76±2.17 | 43.16±2.25 | 0.658 | 44.41±2.71 | 45.25±1.66 | 0.780 | 40.65±2.04 | 36.59±2.46 | 0.208 |
| % intraoperative blood loss | 63.99±8.09 | 59.06±8.30 | 0.673 | 49.29±7.45 | 61.19±6.65 | 0.250 | 108.92±13.01 | 131.33±19.13 | 0.322 |
| % total blood loss | 91.32±10.49 | 77.11±11.46 | 0.365 | 69.75±8.99 | 78.68±8.72 | 0.495 | 141.34±18.75 | 155.96±24.21 | 0.630 |
| Cobb angle（°） | 68.57±3.19 | 57.11±3.01 | 0.013 | 93.25±1.56 | 90.42±1.23 | 0.159 | 119.57±3.28 | 127.59±4.16 | 0.133 |
| amount of blood recovered with the cell saver |  | 485.32±75.71 |  |  | 518.71±71.31 |  |  | 707.53±124.15 |  |
| % of lost blood recovered with the cell saver |  | 16.40±2.47 |  |  | 16.93±0.226 |  |  | 29.91±5.82 |  |
| $ total cost (China standard) | 777.66±64.44 | 887.27±89.28 | 0.319 | 667.85±79.04 | 977.81±81.88 | 0.013 | 1171.56±155.55 | 1353.92±116.05 | 0.384 |
| $ total cost (US standard) | 1332.14±112.91 | 1295.21±159.84 | 0.849 | 1131.25±134.69 | 1449.02±144.78 | 0.137 | 1998.91±292.72 | 2106.59±200.92 | 0.780 |
| Intraoperative EBL (ml) | 1730.95±178.44 | 1776.32±271.51 | 0.888 | 1487.50±208.14 | 1916.67±220.97 | 0.188 | 2956.52±317.09 | 3023.53±348.69 | 0.889 |
| Total perioperative amount of EBL (ml) | 2468.10±217.95 | 2296.32±364.62 | 0.682 | 2084.38±252.70 | 2471.25±285.60 | 0.348 | 3833.70±431.73 | 3559.82±432.84 | 0.664 |
